# β-Cyclodextrin-Based Nanosponges Inclusion Compounds Associated with Gold Nanorods for Potential NIR-II Drug Delivery

**DOI:** 10.3390/pharmaceutics14102206

**Published:** 2022-10-17

**Authors:** Sebastián Salazar Sandoval, Elizabeth Cortés-Adasme, Eduardo Gallardo-Toledo, Ingrid Araya, Freddy Celis, Nicolás Yutronic, Paul Jara, Marcelo J. Kogan

**Affiliations:** 1 Departamento de Química, Facultad de Ciencias, Universidad de Chile, Las Palmeras 3425, Ñuñoa, Santiago 7800003, Chile; 2 Departamento de Química Farmacológica y Toxicológica, Universidad de Chile, Sergio Livingstone 1007, Santiago 8380492, Chile; 3 Advanced Center for Chronic Diseases (ACCDiS), Universidad de Chile, Santos Dumont 964, Independencia, Santiago 8380494, Chile; 4 Laboratorio de Procesos Fotónicos y Electroquímicos, Facultad de Ciencias Naturales y Exactas, Universidad de Playa Ancha, Valparaíso 2360002, Chile; 5 ATMOS-C, Centro de Espectroscopía Atómica y Molecular, Universidad de Playa Ancha, Valparaíso 2360001, Chile

**Keywords:** β-cyclodextrin-based nanosponges, curcumin, melphalan, gold nanorods, photothermal drug release, near-infrared laser light, second biological window, tumor therapy

## Abstract

This article describes the synthesis and characterization of two nanocarriers consisting of β-cyclodextrin-based nanosponges (NSs) inclusion compounds (ICs) and gold nanorods (AuNRs) for potential near-infrared II (NIR-II) drug-delivery systems. These nanosystems sought to improve the stability of two drugs, namely melphalan (MPH) and curcumin (CUR), and to trigger their photothermal release after a laser irradiation stimulus (1064 nm). The inclusion of MPH and CUR inside each NS was confirmed by field emission scanning electron microscopy (FE-SEM), Raman spectroscopy, Fourier transform infrared spectroscopy, (FT-IR) differential scanning calorimetry (DSC), transmission electron microscopy (TEM), and proton nuclear magnetic resonance (^1^H-NMR). Furthermore, the association of AuNRs with both ICs was confirmed by FE-SEM, energy-dispersive spectroscopy (EDS), TEM, dynamic light scattering (DLS), ζ-potential, and UV–Vis. Moreover, the irradiation assays demonstrated the feasibility of the controlled-photothermal drug release of both MPH and CUR in the second biological window (1000–1300 nm). Finally, MTS assays depicted that the inclusion of MPH and CUR inside the cavities of NSs reduces the effects on mitochondrial activity, as compared to that observed in the free drugs. Overall, these results suggest the use of NSs associated with AuNRs as a potential technology of controlled drug delivery in tumor therapy, since they are efficient and non-toxic materials.

## 1. Introduction

β-Cyclodextrins (β-CDs) are water-soluble macrocyclic oligosaccharides, consisting of seven glucopyranose units bonded by an α-1,4 glycosidic linkage. β-CDs stand out because of their cavity dimensions (7.8 A), which can form complexes with benzyl compounds.

As such, β-CDs have been widely investigated to increase drug bioavailability due to their well-defined structure, moderate toxicity when administered locally or orally, and their stability with crosslinking agents, such as carbonyl compounds, carboxylic acids, and epoxides to form nano-porous formulations [1,2,3,4,5,6]. In this context, β-CD-based nanosponges (NSs) emerge as carriers with a sponge-like morphology and lipophilic nanochannels, formed through the cross-linking of β-CD monomers. NSs are the material of choice over β-CDs due to their higher stability, biocompatibility, encapsulation efficiencies, and control over their particle size and solubility [7,8,9,10,11]. Moreover, NSs increase the permeability of dermal formulations, control the drug release rate, and lessen drug degradation [12,13,14,15,16,17].

Among the different molecules that have been studied in the context of their potential anti-tumor effects, polyphenols and nitrogen mustards have shown to be effective against various types of tumor cell lines, such as lung, multiple myeloma, ovarian, breast and gastric cancer, among others [18,19,20,21]. Curcumin ((1E,6E)-1,7-bis(4-hydroxy-3-methoxyphenyl)-1,6-heptadiene-3,5-dione) is a naturally occurring polyphenol phytoconstituent obtained from *Curcuma longa*. In recent decades, curcumin (CUR) has received increasing attention due to its biofunctional properties [22,23,24,25]. On the other hand, melphalan (4-[bis(2-chloroethyl)amino] -L-phenylalanine) is a derivative of nitrogen mustard with antineoplastic activity [18,26,27].

Despite their potential beneficial effects, these therapeutic agents have some limitations, which hinder their therapeutic efficacy. Both CUR and melphalan (MPH) exhibit poor bioavailability, very low aqueous solubility and undergo photodegradation. To overcome these limitations, and to minimize potential side-effects, several strategies have been studied, such as the development of liposomal, biopolymeric and polysaccharide-based formulations.

In this scenario, β-CDs-based NSs are an interesting alternative to solve these disadvantages. NSs may form inclusion compounds (ICs) with different molecules, by using the multiple supramolecular sites that are formed in the cross-linking reaction, and can be used as drug-delivery systems, as previously reported [5,28,29,30,31]. In recent years, several studies have shown that the decoration of NSs with metal nanoparticles can improve the usefulness and properties of the polymer, namely magnetite [10,32,33], silver [34,35], or gold nanoparticles [36].

Among the latter, the use of gold nanorods (AuNRs) as potential nanocarriers for controlled drug release has been widely reported [37]. AuNRs show both transversal and longitudinal surface plasmon absorption peaks; while the former generally appear around 530 nm, the latter may appear in the near-infrared (NIR) region, thus creating the possibility of penetrating deep inside biological tissues. Furthermore, since the modification of the AuNRs aspect ratio changes the longitudinal plasmon band’s wavelength, this may be used to shift the plasmon band to the NIR-II region. It is here, in the NIR-II region, or so-called second biological window (1000–1300 nm), where AuNRs have proven to have better tissue penetration, low photon scatter, low background signal and higher allowable exposure with respect to the NIR-I region (650–950 nm) [38,39,40,41]. Therefore, AuNRs may be irradiated to generate localized heat in their proximity and trigger the controlled release of drugs by means of local photothermia. Taking this into account, AuNRs might be associated with inclusion complexes (Ics) if the guests present functional groups, such as thiols, amines, or hydroxyls. NSs associated with AuNRs might promote the release of the included guests by means of NIR-II, due to the plasmon effect of AuNRs.

**Figure 1:**
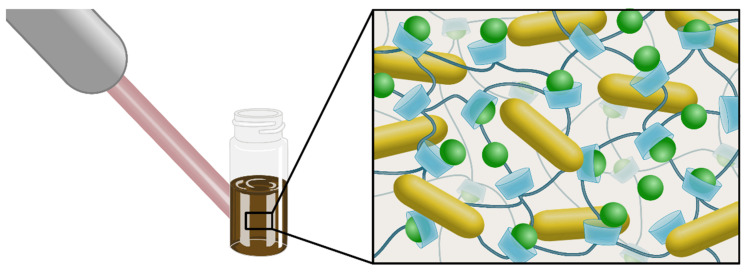
Schematic representation of the NSs–drug complexes associated with AuNRs. Laser light stimuli of 1064 nm is absorbed by AuNRs and transformed into local heat, which induces the release of MPH and CUR (represented in green circles) from the cavities of NSs.

This report describes the inclusion complexes of NSs–CUR and NSs–MPH associated with AuNRs, with plasmon centered at the NIR-II region (Figure 1). The inclusion of MPH and CUR inside each NS was confirmed using a battery of physicochemical characterizations, and the association of AuNRs with both ICs was also analyzed.

The drug release of both guests via plasmonic photothermia was assayed using a continuous laser irradiation stimuli of 1064 nm, which showed that the controlled-photothermal drug release of both MPH and CUR inside the cavities of NSs reduces the cytotoxic effect as compared to that of free drugs. To the best of our knowledge, a controlled drug-delivery system consisting of AuNRs and NSs ICs has not been reported to date. Our results show that NSs–AuNRs complexes are efficient and non-toxic materials that might eventually be considered as a potential technology for tumor therapy.

## 2. Methods

### 2.1. Materials

Anhydrous β-cyclodextrin, C_42_H_70_O_35_, ≥97%, 1134.98 g/mol; melphalan, C_13_H_18_Cl_2_N_2_O_2_, ≥90%, 305.2 g/mol; curcumin, C_21_H_20_O_6_, 99%; diphenyl carbonate, C_6_H_5_O, 99%, 214.2 g/mol; tetrachloroauric acid, HAuCl_4_, ≥99.9%, 339.7 g/mol; polyethylene glycol (PEG), H(OCH_2_CH_2_)nOH; sodium hydroxide, NaOH, ≥99%, 39.9 g/mol; cetyltrimethylammonium bromide (CTAB), C_19_H_42_BrN ≥ 98%, 364.45 g/mol; hydroquinone, C_6_H_6_O_2_, ≥99%, 110.11 g/mol; sodium borohydride, NaBH_4_, ≥99%, 37.83 g/mol, and nano-pure water are commercially available from Merck (Merck, Darmstadt, Germany). All glassware was washed thoroughly with aqua regia (3 HCl:1 HNO_3_) and Milli-Q water.

### 2.2. Synthesis of AuNRs

The AuNRs were synthesized using a modified seed-mediated method [42]. The seed was prepared by mixing 4.915 mL of CTAB (0.1 M) with 85 μL of HAuCl_4_ (29.4 mM) on a small flask with constant stirring for 5 min; then, 460 μL of NaBH_4_/NaOH solution was added (10 mM/0.01 M) to the mix and stirred for 0.5 h. Preparation of the growth solution was performed by adding 170 μL of HAuCl_4_ (29.4 mM) to 9.83 mL of a CTAB solution (0.1 M) and stirring for 10 min. Then, 1000 μL of AgNO_3_ (10 mM) were added to the mixed solution, and, after stirring for 30 s, 500 μL of hydroquinone (100 mM) were added, stirring for another 30 s. Finally, 160 μL of the seed solution were mixed with the growth solution, stirred for 30 s, and left to rest overnight.

The AuNRs were centrifuged, resuspended in Milli-Q water, and stabilized with PEG to remove the CTAB from the nanoparticles. The synthesis was carried out at 27 °C on a water bath to prevent the CTAB from crystallizing.

### 2.3. Synthesis of the NSs

**Figure 2:**
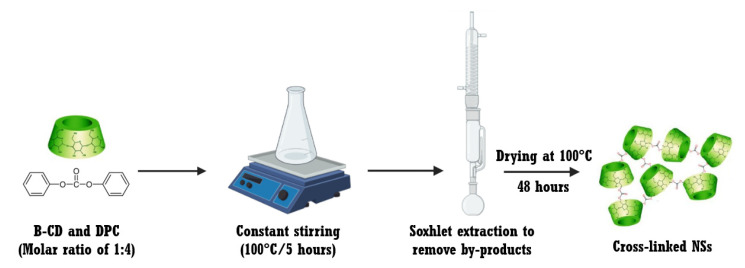
Schematic representation of the synthetic route of NSs.

NSs were synthesized with minor modifications from previously reported methods [10,43], using β-CD and diphenyl carbonate (DPC) as precursors. Anhydrous β-CD (1.5 g) and DPC (0.856 g) were homogenized in a solid state, placed in a conical flask, and heated from 90 to 100 °C under constant stirring for 5 h. The reaction mixture was left at room temperature until it cooled down, and the obtained solid was ground with an agate mortar. Double distilled water and acetone separated the product from the unreacted precursors. Afterward, the solid was washed with Soxhlet extraction with ethanol and acetone for 48 h to remove phenol, which formed as a by-product of the cross-linking reaction. Finally, the solid was dried at 100 °C for 48 h and stored at room temperature for further use. Figure 2 illustrates the synthetic route of NSs.

### 2.4. Preparation of NSs–MPH and NSs–CUR Complexes

Each compound, namely, MPH and CUR, were loaded into the cavities of NSs using reported methods [29,44,45]. A total of 20 mg of NSs were immersed in 50 mL of double-distilled water and kept under constant agitation. Afterward, 20 mL of MPH 0.1 mM or CUR 0.1 mM solution were added to the dispersed NSs. Both mixtures were sonicated for 10 min. and left under constant stirring for 1 day. The uncomplexed drugs were separated from the suspensions using centrifugation at 3000 rpm for 30 min. The obtained supernatants were freeze-dried at −81 °C and 0.001 mbar. The dried powders corresponding to the NSs–MPH and NSs–CUR complexes were stored in a desiccator for further use.

### 2.5. Association of AuNRs into the ICs

The association of AuNRs with the NSs–MPH or NSs–CUR complexes was carried out by immersing 30 mg of the ICs in 0.5 mL of AuNRs. After settling for 20 min., the mixture was centrifuged at 20.000 rpm for 30 min. The AuNRs associated with the ICs were separated from the supernatant and dried under vacuum. AuNRs concentration after association with the NSs drug complexes was determined using UV-Visible spectroscopy.

### 2.6. Proton Nuclear Magnetic Resonance (^1^H-NMR) Spectroscopy

^1^H-NMR characterization was performed using a Bruker Advance 400 MHz spectrometer (Bruker, Billerica, MA, USA) at 30 °C. Tetramethyl silane (TMS) was used as an internal standard. Stock solutions of NSs, the drugs, and the ICs were prepared using deuterated dimethyl sulfoxide (DMSO)-d_6_ as solvent due to the low solubility of NSs in deuterated water/chloroform, as reported previously [13,46,47,48]. Data processing was carried out using the Mestre nova program.

### 2.7. Field Emission Scanning Electron Microscopy (FE-SEM)

The surface morphology features of NSs, MPH, CUR, and the ICs were analyzed using a Zeiss LEO Supra 35-VP scanning electron microscope equipped with EDS. Acceleration voltages of 2.0 and 5.0 kV were used. The samples were deposited onto a carbon tape stuck to an aluminum stub, following gold coating using a magneton sputtering (pressure of 0.5 mbar, argon atmosphere, and current of 25 mA, for 15 s) to minimize charging effects.

### 2.8. Ultraviolet and Visible Absorption (UV–Vis) Spectroscopy

UV-Visible spectra of the AuNRs and the ICs associated with AuNRs were measured using a Jasco V-760 UV-Visible spectrometer. Measurements were conducted in the range of 200–1100 nm, using deionized water as a reference. The UVProve 1.10 program was used for data processing.

### 2.9. Raman Spectroscopy

Raman spectra of the samples were acquired using a WI Tec SNOM/Raman microscopy model Alpha 300 equipped with a 785 nm laser line and employing the 50× objective. The Raman spectra (200–1700 cm^−1^) of the samples were registered setting the conditions as follows: 10 acquisitions with 10 s of integration time per spectrum. the intrinsic fluorescence of samples was quenched by using a thin sheet of gold prepared by metal sputtering method.

### 2.10. Fourier Transform Infrared Spectroscopy (FT-IR)

FT-IR spectra of the samples were acquired using a Jasco spectrometer model 4600 equipped with a Deuterated L-alanine Doped Triglycine Sulphate (DLATGS) detector. A total of 150 scans per sample (400–4000 cm^−1^) were performed by placing each sample on a micro-ATR (ATR pro one) accessory using a ZnSe crystal.

### 2.11. Transmission Electron Microscopy (TEM)

TEM analyses of AuNRs, NSs, ICs, and the ICs associated with the AuNRs were performed using a Hitachi model HT-7700 microscope, operating at 120 kV. The ICs associated with AuNRs were dispersed in ethanol (30% *v*/*v*). After sonication for 5 min., 10 µL of the formulations were deposited onto a copper grid with a Formvar film. In the case of AuNRs, 10 µL was deposited directly on a copper grid with a Formvar film. All samples were dried overnight for resolution enhancement.

### 2.12. Differential Scanning Calorimetry (DSC)

MPH, CUR, NSs, NSs–MPH, and NSs–CUR complexes were analyzed on a Differential Scanning Calorimeter DSC 8000 Perkin Elmer to obtain their respective DSC thermograms. Aluminum pans were used to place, weigh, and seal the samples. Measurements were carried out over a temperature range of 0–600 °C under a continuous nitrogen flow rate of 10 °C/min.

### 2.13. Determination of Drug Content in NSs

The encapsulation efficiency (EE) of NSs–MPH and NSs–CUR complexes can be defined as the concentration of the complexed drug over the initial concentration of the drug. EE values were obtained using Equation (1), as follows:

(1) $$\mathrm{EE}\;\left(\%\right)=\frac{\left[\mathrm{Drug}\right]\;\mathrm{in}\;\mathrm{NSs}}{\mathrm{initial}\;\left[\mathrm{Drug}\right]}\times 100\%$$

The loading capacity (LC) of NSs–MPH and NSs–CUR was obtained from the total weight of NSs and the weight of entrapped drugs using Equation (2):

(2) $$\;\mathrm{LC}\;\left(\%\right)=\frac{\mathrm{Drug}\;\mathrm{weight}\;\mathrm{in}\;\mathrm{NSs}}{\mathrm{Weight}\;\mathrm{of}\;\mathrm{NSs}}\times 100\%$$

### 2.14. DLS and ζ-Potential

Size distribution, polydispersity index, and ζ-potentials were determined using a DLS Zetasizer NanoS series, Malvern. Proper dilution of all samples with double-distilled water was carried out before measurements were performed at 25 °C using disposable zeta cells. The size distribution and ζ-potentials were calculated using the cumulants fit and the Smoluchowsky approximation, respectively. A total of 12 measurements were acquired, expressing the results as their average. For NSs–based samples, measurement conditions were set as follows: refraction index: 1.49, k:0; whereas, for AuNRs, measurements were performed using a refraction index of 1.33 and k:0.

### 2.15. Laser Irradiation Assays

For laser irradiation assays, a laser at 1064 nm, with a light power of 150 mW and beam diameter of 1 mm, was used. A total of 200 µL of the NSs–MPH and NSs–CUR complexes conjugated with the AuNRs were added to a 500 µL Kahn test tube. The ternary systems were exposed to laser irradiation at different times (intervals of 1 min until reaching a maximum of 20 min). The release of both MPH and CUR was measured using UV-Vis spectroscopy. ICs without AuNRs were irradiated for control assays to determine whether the AuNRs were responsible for the release of the guest molecules.

Maximum absorbances of the released drugs were expressed as release percentages and then compared with the initial amount of the drug. All assays were carried out in triplicate. Percentages of the released drug (DR) were calculated using the following Equation (3):

(3) $$\;\mathrm{DR}\;\left(\%\right)=\frac{\mathrm{released}\;\left[\mathrm{Drug}\right]\;}{\;\mathrm{initial}\;\left[\mathrm{Drug}\right]}\times 100\%$$

### 2.16. Mitochondrial Activity Assays

Mitochondrial activity was measured by MTS using the CellTiter 96 AQueous one solution cell proliferation assay (Promega). The experiments were conducted as recommended by the manufacturer. In brief, 5000 cells per well were seeded on 96-well plates in 100 µL of complete Dulbecco’s Modified Eagle Medium (DMEM). The medium was incubated at 37 °C and was subsequently removed after 1 day. Further, cells were incubated for another day with a titration (1 to 1 serial dilutions) of MPH, CUR, NSs–MPH, and NSs–CUR (all samples ranging from 0.1, 0.05, 0.025, and 0.01 mM in 1% DMSO, and then the volume was completed with DMEM medium). Afterward, phenol red-free DMEM medium (Gibco) containing the MTS/PMS reagent was added to replace the medium and incubated for 1 h at 37 °C. Absorbance measurements of all samples were carried out with a microplate reader at 490/655 nm (Synergy Mx, Biotek). For each experiment, fluorescence was corrected by subtracting the average fluorescence from a triplicate set of control wells without cells. Mitochondrial activity was calculated with respect to a non-treated control (medium). Each experiment was performed in 3 technical and 2 biological replicates.

### 2.17. Data Analysis

All the results are presented as mean ± SD, determined by at least three independent experiments. Statistical analyses were conducted using GraphPad Prism 9 Software Inc. (San Diego, CA, USA). A one-way ANOVA, followed by Tukey’s Test, was performed to determine significance between results, which were considered as such if **** *p* < 0.0001, *** *p* < 0.001, and * *p* < 0.05.

## 3. Results and Discussion

### 3.1. Characterization of the ICs

#### 3.1.1. ^1^H-NMR Spectra of the ICs

**Figure 3:**
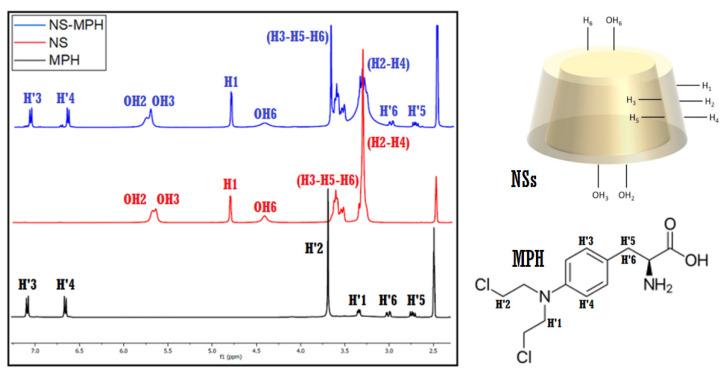
^1^H-NMR spectra (400 MHz, DMSO-d_6_) of MPH, NSs, and NSs–MPH.

**Figure 4:**
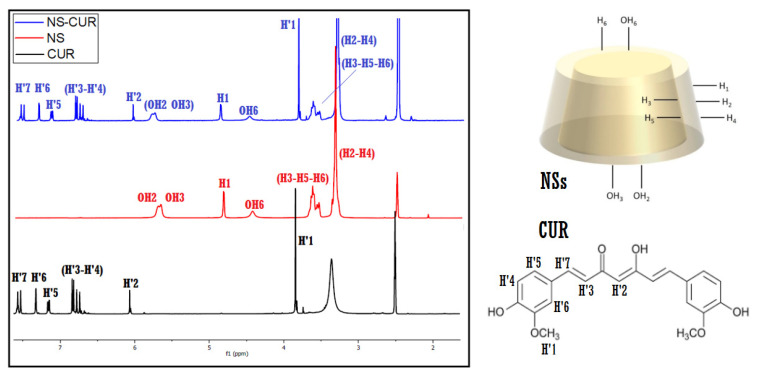
^1^H-NMR spectra (400 MHz, DMSO-d_6_) of CUR, NSs, and NSs–CUR.

The inclusion of MPH and CUR inside the cavities of the NSs was confirmed using ^1^H-NMR spectroscopy. The changes in the chemical shifts in the protons of both NSs and the drugs provided evidence for the formation of the NSs–MPH and NSs–CUR complexes. The acquired spectra of NSs, MPH, CUR, and the ICs are shown in Figure 3 (adapted from [36]) and Figure 4.

Proton signals of both guest molecules showed high-field chemical shifts, possibly due to screening effects caused by the change in the environment of the drugs, as they ended up entrapped inside the multiple interstitial sites of the NSs. The spatial restriction of MPH and CUR also contributed to the chemical shifts shown by the protons of both guests.

Notably, the protons within the hydrophobic cavities of the NSs (H3, H5 and H6) and the OH2 and OH3 hydroxyl groups displayed the most pronounced chemical shifts among the protons of NSs, which strongly suggests complexation of the drugs. Chemical shifts can also be observed for the protons located in the external cavities of the NSs (H1, H2, H4), implying that the complexation of MPH and CUR occurs in both the cavities of the β-CD monomers and the supramolecular sites that are produced in the polymerization, in accordance with previous studies of NSs inclusion compounds [10,36,49,50,51,52]. The largest chemical shifts in both drug molecules correspond to the protons present in the benzyl ring structure, indicating their preferential inclusion inside the multiple β-CD cavities of NSs. Chemical shifts in MPH and CUR before and after inclusion are shown in Table 1 (adapted from [36]) and Table 2, respectively.

#### 3.1.2. FE-SEM Analyses of the ICs

**Figure 5:**
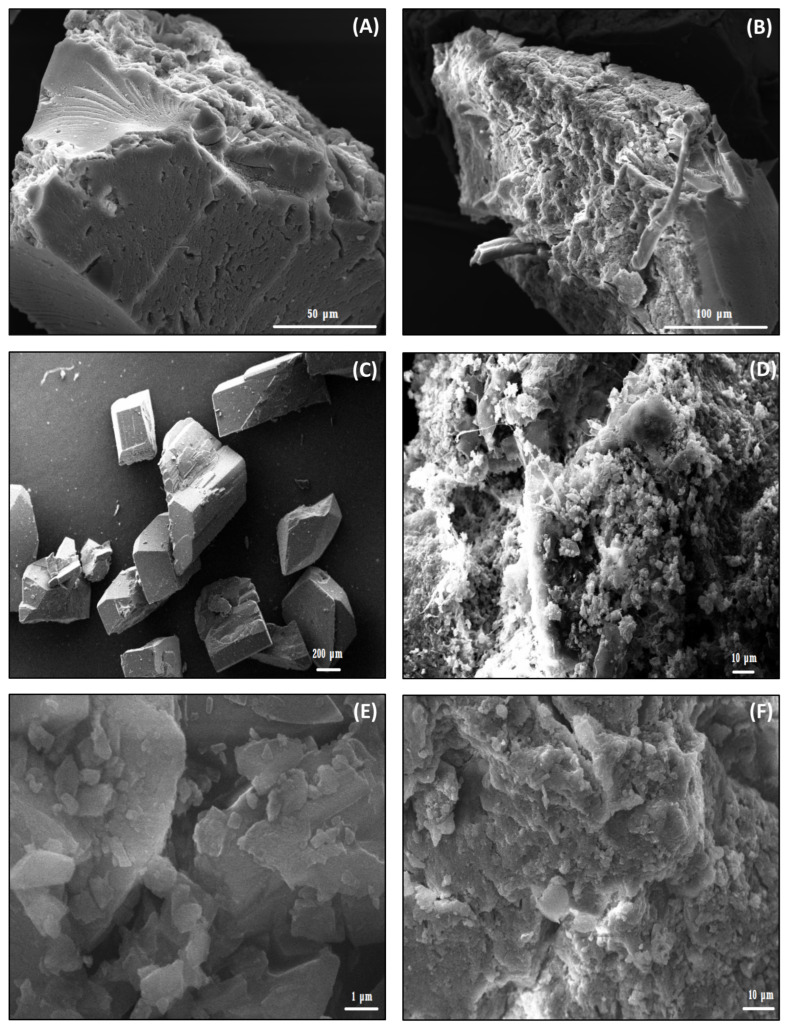
FE-SEM images of NSs (**A**,**B**), MPH (**C**), NSs–MPH (**D**), CUR (**E**), and NSs–CUR (**F**).

The formation of the NSs–MPH and NSs–CUR complexes can also be confirmed by FE-SEM analyses. Figure 5 shows SEM micrographs of NSs, MPH, CUR, and the ICs.

FE-SEM images of NSs confirm their highly rough surface and sponge-like morphology, which might be suitable for the inclusion of the guest molecules, in agreement with previous studies [13,29,32,36,53]. Both MPH and CUR show crystalline morphology. After drug loading, the ICs maintain the morphological features of native NSs, suggesting the formation of an inclusion compound rather than a physical mixture, as co-precipitation of the free drugs was not observed.

#### 3.1.3. TEM Analysis of the ICs

**Figure 6:**
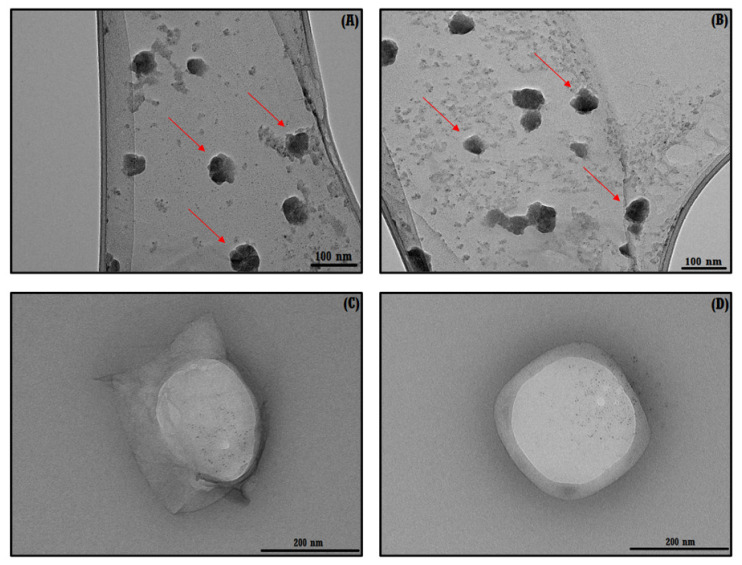
TEM images of NSs (**A**,**B**), NSs–MPH (**C**), and NSs–CUR (**D**). The red arrows (**A**,**B**) highlight some of the synthesized NSs.

The morphology of free NSs, NSs–MPH and NSs–CUR can be elucidated using TEM analyses. As seen in Figure 6A,B, NSs have a spherical nature, with an average size of 90 nm. The NSs–MPH and NSs–CUR complexes also show a spherical structure, and an increase in size to 150 nm after drug loading, with respect to native NSs. This could probably be attributed to intermolecular interactions occurring in the NSs–drug complexes, as reported in previous studies [33,46,47].

#### 3.1.4. Raman and FT-IR Spectra of the ICs

**Figure 7:**
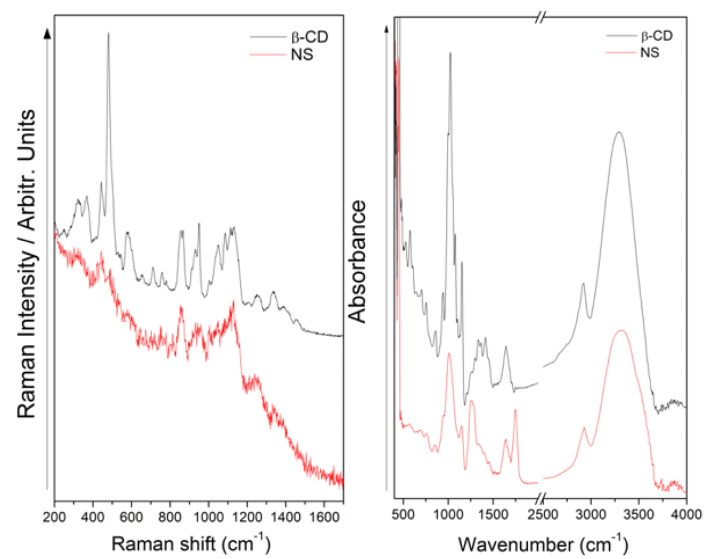
Raman (**left**) and FT-IR spectra (**right**) of β-CD and NSs.

The obtained NSs were characterized by Raman and FT-IR spectroscopy, as can be seen in Figure 7, and the assignment and discussion of the signals were determined based on related published data [10,54,55,56,57]. Spectrums were compared to observe their changes when the NSs were obtained.

In the Raman spectral comparison, the disappearance and decrease in the relative intensity of some bands can be observed in the NSs profile, as a direct consequence of obtaining nanoparticles. The decrease in the relative intensity of the signal observed at 478 cm^−1^ in the NS profile allowed for us to infer a vibration of the compound’s skeletal structure, which was now restricted because of the new structure; the same can be stated for the signal observed at 950 cm^−1^. Related to this, the 576 cm^−1^ band is absent in the Raman profile of NSs, being consistent with the observed spectral facts explained above. Finally, significant spectral data were observed in the FT-IR spectrum of NSs as a new band at 1755 cm^−1^, attributed to the C=O vibration. This signal confirms that we obtained the nanosponge because of the presence of the C=O stretching from the linker.

**Figure 8:**
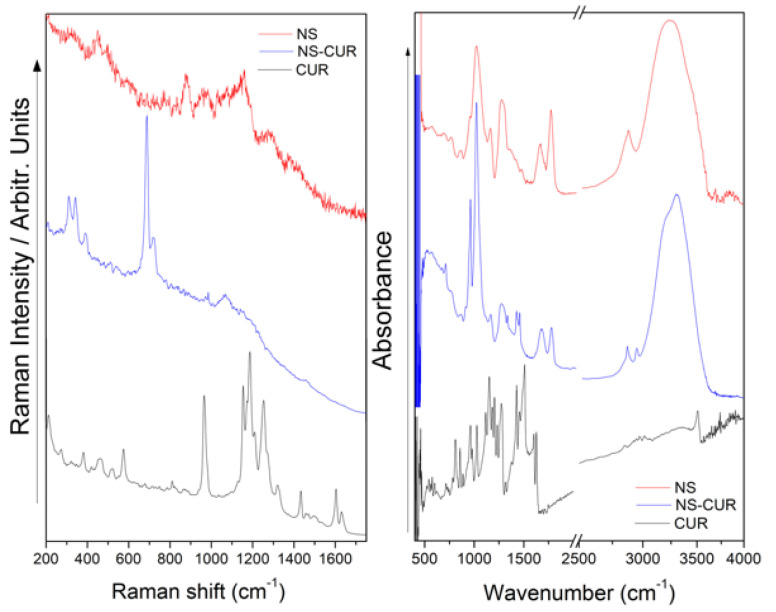
Raman (**left**) and FT-IR spectra (**right**) of NSs, CUR and NSs–CUR.

The ICs systems were also characterized by Raman and FT-IR. In this case, it is important to mention that FT-IR spectra gave us more molecular information than Raman because the latter has a low cross-section, even more so considering the low molar concentration of the CUR and MPH in each system. In the Raman profile of NSs–CUR (see Figure 8), a strong band observed at 672 cm^−1^ and another signal with medium relative intensity located at 703 cm^−1^ were ascribed to an out-of-plane deformation of the ring in CUR. However, the preparation of the NSs–CUR system was confirmed by the FT-IR spectrum (see Figure 8) from the signals observed at 1446, 1408, and 949 cm^−1^, ascribed to in-plane deformation of −CH_3_ groups, stretching of C=CH, and in-plane deformation of the ring, respectively. Furthermore, the interaction of CUR in the NSs is supported by additional signals observed in the FT-IR spectrum at 2912, 3009, 3263, and 3378 cm^−1^.

**Figure 9:**
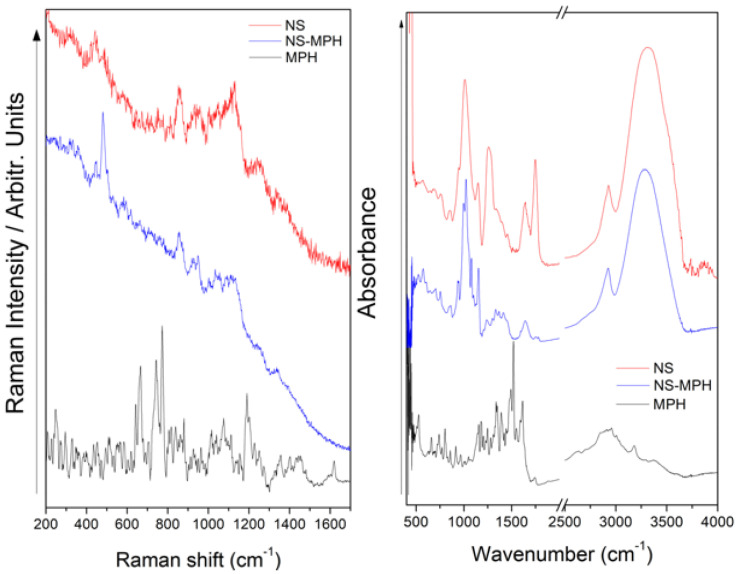
Raman (**left**) and FT-IR spectra (**right**) of NSs, MPH and NSs–MPH.

Finally, the Raman and FT-IR profiles of the NSs–MPH system offered us information about the interaction of the species. The Raman spectrum (see Figure 9) displays two bands at 442 and 481 cm^−1^, corresponding to the NNC and CCN bending mode in MPH, respectively. The FT-IR spectrum of NSs–MPH (see Figure 9) is dominated by the profile of the NSs; however, there are some shifts in specific signals. Some bands observed at 1027, 1099, 1074, 1644 and 1755 cm^−1^ show the mentioned shift because of the interaction between the species. A new band appeared at 1779 cm^−1^ and is ascribed to C=O vibrations from MPH.

#### 3.1.5. DSC Thermograms of the ICs

**Figure 10:**
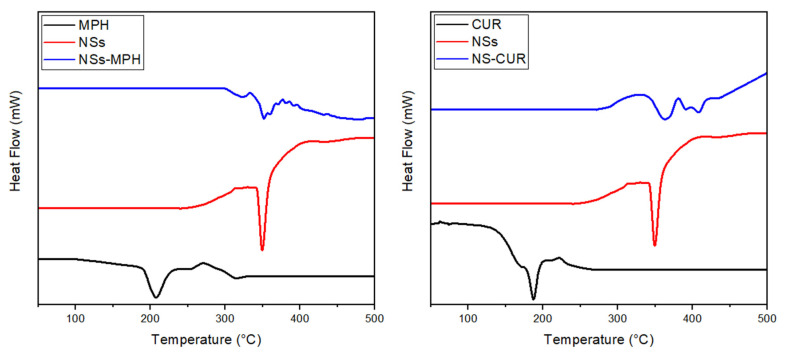
DSC thermograms of MPH, CUR, NSs, NSs–MPH, and NSs–CUR.

The NSs–MPH and NSs–CUR complexes were characterized through DSC. Thermal analyses of NSs, the ICs, and the free drugs were performed to confirm the formation of an inclusion compound between the guests and the NSs matrix rather than a physical mixture. Figure 10 depicts the thermograms of MPH, CUR, NSs, NSs–MPH, and NSs–CUR. NSs exhibit an endothermic peak at 350 °C, representing the melting point of the crosslinked polymer, as reported previously [43,58,59]. DSC thermogram of MPH shows a sharp endothermic peak at around 203 °C, corresponding to the melting point of the free drug [60].

Free CUR showed an endothermic peak at 183 °C, which, according to the literature, corresponds to its intrinsic melting point [25,61]. Furthermore, the thermograms of the ICs indicate the disappearance of the characteristic peaks in the drugs, suggesting the complexation of the guests inside the cavities of NSs, while excluding the possibility of a physical mixture. Similar results have been observed for other NSs–drug complexes [43,45,62,63,64,65,66].

### 3.2. Characterization of ICs Associated with the AuNRs

#### 3.2.1. TEM, UV-Vis, DLS, and ζ-Potential of AuNRs

**Figure 11:**
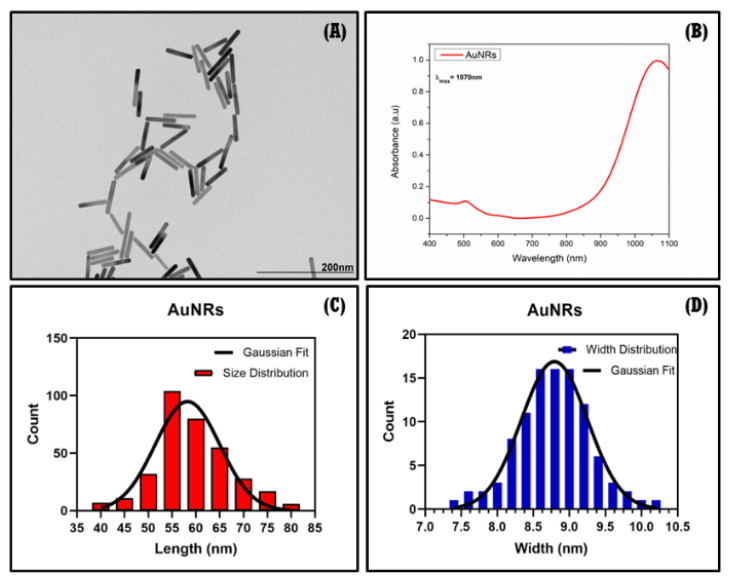
TEM images (**A**), UV-Vis NIR-II spectra (**B**), and size distribution of AuNRs (**C**,**D**).

The characterization of AuNRs was carried out by TEM, UV-Vis, DLS and **ζ**-potential. Figure 11A shows the TEM micrograph of the AuNRs, and its size distribution was estimated. Both the length and width of the nanoparticles were plotted (Figure 11C,D) and their average sizes were 55 nm and 8.6 nm, respectively. The UV-Vis spectra of AuNRs confirmed the presence of its characteristic plasmon band in the NIR-II window, showing a maximum absorbance for the longitudinal plasmon at 1070 nm (Figure 11B), making them suitable for biological and photothermal applications [67]. The aspect ratio (length/width) for the synthesized AuNRs was 6.4. This agrees with the reported values, which indicate that AuNRs absorb in the second biological window [42,68,69].

The hydrodynamic diameter (D_h_) of AuNRs provided by DLS is shown in Table 3, along with the **ζ**-potential. The D_h_ was 9.02 ± 4.1 nm and 84.7 ± 46.7 nm, which can be attributed to the rotational and translational light dispersed by the nanoparticles, respectively. The polidispersity index (PDI) was 0.47, thus indicating that the nanoparticles exhibited good monodispersity (PDI < 0.7). Furthermore, the **ζ**-potential was estimated to be −30 ± 3.9 mV, suggesting good colloidal stability and that the AuNRs would not undergo aggregation over time.

#### 3.2.2. FE-SEM and EDS Mapping Analyses of the ICs Associated with the AuNRs

**Figure 12:**
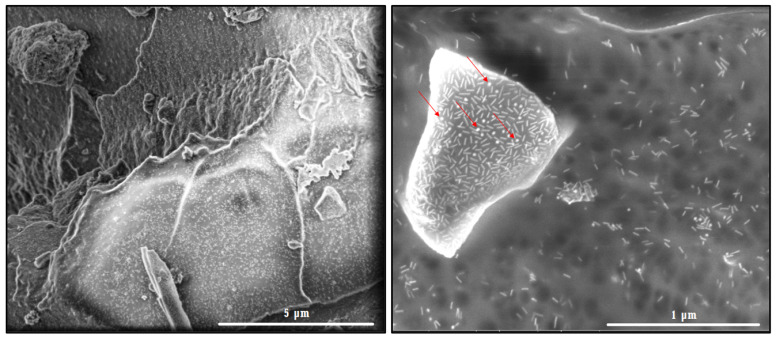
FE-SEM of the ICs associated with the AuNRs. The red arrows (right panel) highlight the impurities ascribed to spherical gold nanoparticles.

The AuNRs attached to the ICs are shown in Figure 12. The ICs retained their porous morphology after their association with the AuNRs. The FE-SEM images indicate that the AuNRs are homogeneously distributed all over the organic matrix, with no evident changes in their aspect ratio nor morphology, thus confirming that the NSs–drug complexes are optimum substrates for stabilizing the AuNRs.

The micrographs also show a third component in the ICs, corresponding to spherical gold nanoparticles, which constitutes an inherent impurity of AuNRs synthesis.

**Figure 13:**
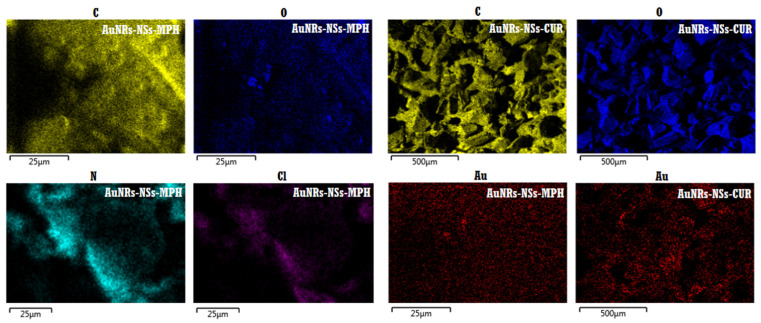
EDS mapping analyses of AuNRs-NSs–MPH and AuNRs-NSs–CUR.

EDS analysis provided information of the ICs associated with the AuNRs regarding their elemental composition, as seen on Figure 13. The elemental mapping shows the presence of C, O, N, and Cl in the AuNRs–NSs–MPH complex, where N and Cl can be attributed to the amine and chloroethylamine functional groups of MPH. Elemental mapping of AuNRs–NSs–CUR evidenced the presence of C and O on the supramolecular matrix, corresponding to the functional groups of both NSs and CUR. The EDS analyses also showed the detection of Au, thus confirming the association of AuNRs and the ICs.

#### 3.2.3. TEM Analyses of the ICs Associated with the AuNRs

**Figure 14:**
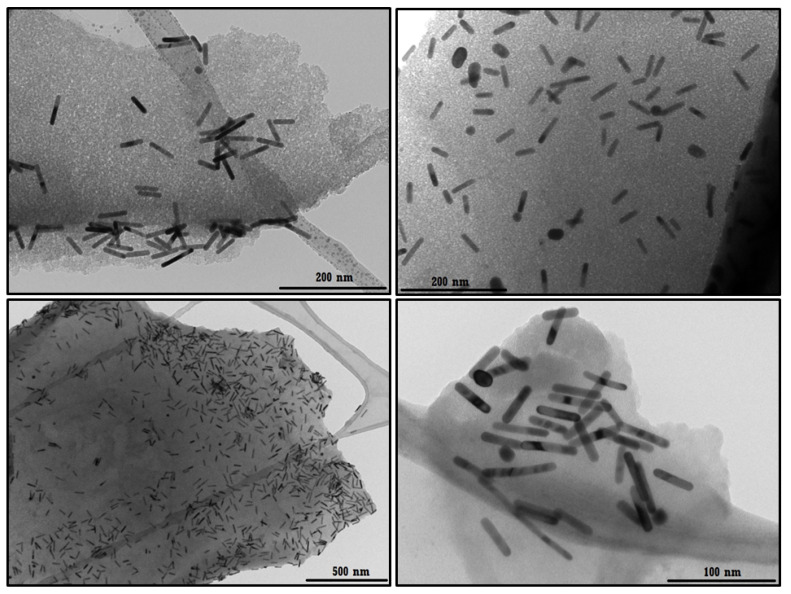
TEM images of the ICs associated with AuNRs.

TEM micrographs of the ICs associated with AuNRs are shown in Figure 14. Immobilization of AuNRs in the polymeric matrix does not seem to affect their integrity and aspect ratio, indicating that the formation of the AuNRs-NSs–MPH and AuNRs-NSs–CUR systems contribute to the nanoparticles’ stability, which is consistent with the FE-SEM images shown in Section 3.2.2. The micrographs also show that the impurities assigned to spherical gold nanoparticles are minimal.

#### 3.2.4. UV-Vis Spectra of the ICs Associated with the AuNRs

**Figure 15:**
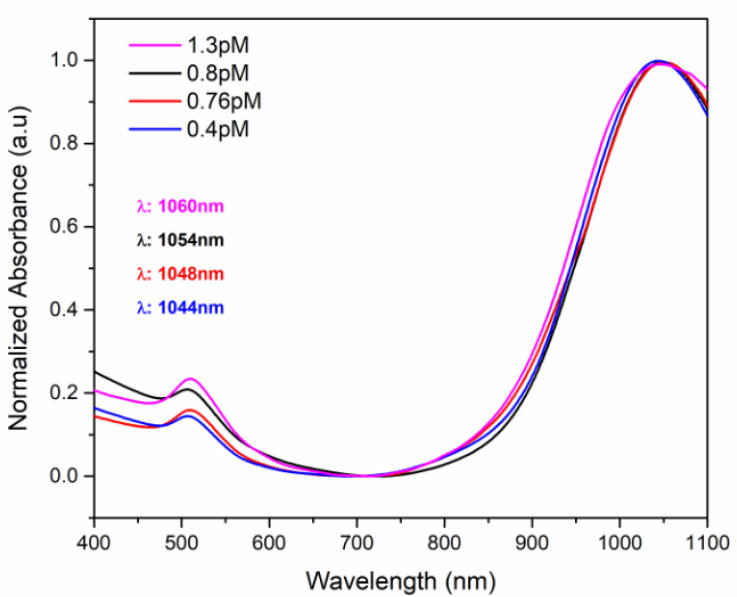
UV-Vis NIR-II spectra of the ICs associated with the AuNRs.

Deposition of AuNRs in the NSs–drug complex can also be confirmed using UV-Vis spectroscopy. Absorption UV-Vis spectra of the ICs associated with AuNRs are shown in Figure 15. The characteristic plasmonic bands of AuNRs were observed at 520 nm and 1060 nm, which correspond to the transversal and longitudinal absorption peaks, respectively.

A hypsochromic shift in the plasmon bands from 1070 nm to 1060 nm was observed, due to the proximity and environmental changes around the AuNRs, after their deposition in the NSs–drug complexes. Notably, the shift of the plasmon resonance peaks increases when the AuNRs concentration decreases. The presence of the plasmonic bands in the ICs-AuNRs provided evidence that the polymeric matrix provides stability to the nanoparticles, as reported by previous studies [36,70,71].

#### 3.2.5. DLS and ζ-Potential of the ICs Associated with the AuNRs

Table 4 depicts the hydrodynamic diameters (DLS), ζ-potentials, and polydispersity indexes (PDI) of AuNRs, the NSs–drug complexes and the ICs associated with AuNRs.

**Figure A1:**
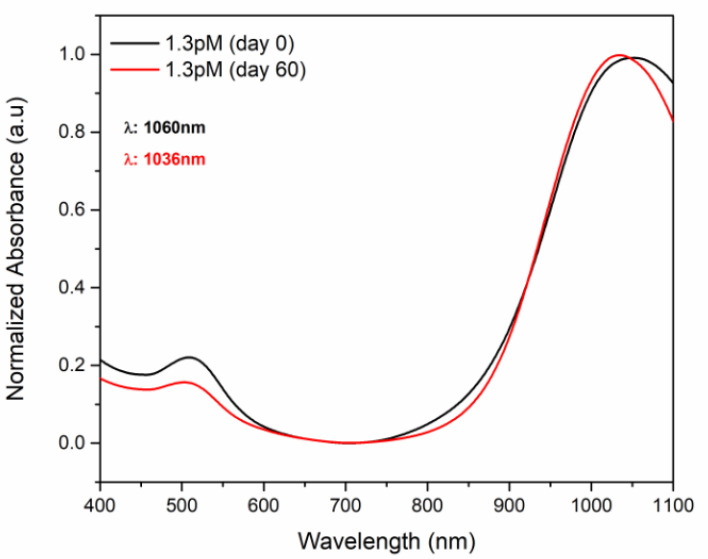
UV-Vis NIR-II spectra of the IC-AuNRs following storage at 4 °C for 2 months.

DLS provided information about the hydrodynamic diameters of the ICs and the ICs-AuNRs. The NSs, NSs–MPH, NSs–CUR and the ternary systems depicted values over 200 nm, thus confirming the nanometric size of the supramolecular systems. Upon immobilization with the ICs, the ζ-potential of the AuNRs changed due to their stabilization by the NSs–drug complexes. All nano-formulations showed ζ-potentials ranging from −21 to −35, confirming their stability (for further information see Figure A1 and Table A1). The PDI values of all samples indicated that the NSs, the NSs complexes, and the ternary systems are stable and homogeneous in nature (PDI < 0.7).

### 3.3. Guest Photothermal Release by Laser Irradiation

#### 3.3.1. Encapsulation Efficiencies and Loading Capacities

The encapsulation efficiencies (EE%) and loading capacities (LC%) of the NSs–MPH and NSs–CUR complexes were determined using Equations (1) and (2), respectively. As described in Table 5, MPH showed a higher encapsulation efficiency and loading capacity than CUR, indicating that the structure of the guest strongly influences the complexation efficiency and molecular binding [72]. This suggests that MPH might be more aptly sized than CUR to be included in the supramolecular sites of NSs. The encapsulation efficiency and loading capacity values for NSs–CUR and NSs–MPH complexes increased, compared to native β-CD, where the calculated values were 30% (EE%) and 20% (LC%) for β-CD–CUR [65,73], and 70% (EE%) and 61% (LC) for β–CD–MPH [74].

#### 3.3.2. Laser Irradiation Assays

**Figure 16:**
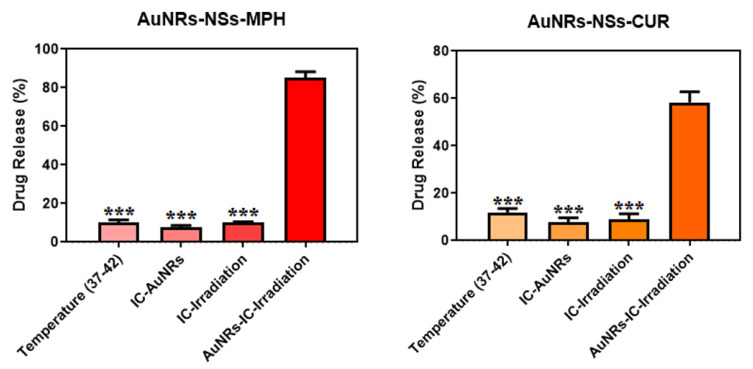
MPH and CUR drug release percentages after 20 min of irradiation. The results are expressed as percentages, compared to the systems used as control, and represent the mean ± SD of *n* = 3 (*** *p* < 0.001).

The ICs were associated with the AuNRs to form a ternary system capable of inducing the release of both MPH and CUR by means of local photothermia using NIR-II irradiation. To achieve this objective, drug released phenomena were studied by adding the ICs conjugated to the AuNRs in a Kahn test tube. Then, the systems were irradiated using a continuous laser of 1064 nm for 20 min. The drug release percentages (%) were determined using the Lambert-Beer equation and Equation (3). The molar attenuation (ε) of both drugs was calculated with UV-Vis spectroscopy using a set of MPH and CUR solutions. Molar attenuation was 10.15 mM^−1^ cm^−1^ for MPH and 4.37 mM^−1^ cm^.1^ for CUR. Drug release percentages in the ternary systems were compared with those calculated in the control systems: ICs without AuNRs, ICs-AuNRs without irradiation, and physiological temperature (37–42 °C) to determine if the guests migrated from the supramolecular sites through diffusion. Figure 16 shows the percentages of released drug at 20 min.

After irradiation, the ternary systems showed the highest drug release percentages of MPH and CUR (about 80% and 60%, respectively). In contrast, the amount of drug released from the AuNRs-ICs at physiological temperature (37–42 °C) was less than 10%. AuNRs exhibit high photothermal efficiencies and effectively diffuse heat to the surrounding media upon exposure to a laser tunable with the AuNRs NIR-II surface plasmon resonance [42,67,68,75]. AuNRs absorb photons, which exchange energy in the metal lattice through electron–phonon coupling and phonon-to-phonon relaxation. This produces an increase in the temperature of the metal surface while also increasing the surrounding local temperature [37,38,42,67]. Thus, the 1064 nm-red laser, with a light power of 150 mW, generated enough local heat in the supramolecular systems to disassemble the AuNRs-ICs complexes and, subsequentially, trigger drug release from the NSs cavities. This confirms that the local photothermal effect produced by AuNRs in the NIR-II window promotes the release of both guests. On the other hand, the drug release percentages were drastically reduced in the control systems, compared to the ICs–AuNRs systems after irradiation. Both anti-tumor drugs migrated from all systems by means of passive diffusion. In summary, the irradiation of the ICs–AuNRs systems allowed a faster and more efficient release of the guests, proving that NIR-II irradiation of the ternary systems may open many opportunities for biological and tumor therapy applications.

### 3.4. MTS Assays

MTS assays were conducted to evaluate the effects of MPH, CUR and the NSs–MPH and NSs–CUR complexes on mitochondrial activity. The aim of this experiment was to evaluate if the inclusion of the drugs inside the NSs reduced their effects on cell viability, since the formulations should not produce cellular toxic effects if they are to be used as therapeutic agents.

**Figure 17:**
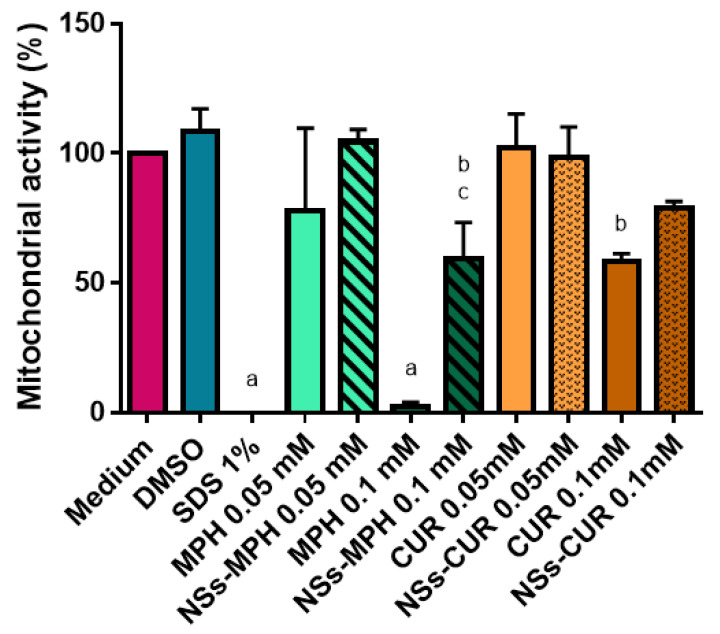
Effects of MPH, CUR, NSs–MPH and NSs–CUR on mitochondrial activity, determined with the MTS assay. The results are expressed as percentages compared with untreated cells (medium) and represent the mean ± SD of *n* = 3 (a = **** *p* < 0.0001, b = * *p* < 0.05). c represents the comparison between MPH 0.1 mM and NSs–MPH 0.1 mM (c = *** *p* < 0.001).

The effects of MPH, CUR, and their corresponding ICs on the mitochondrial activity of HeLa cells were compared at equivalent concentrations (Figure 17). In the case of MPH, both the free drug and its ICs did not show significant effects up to 0.05 mM of drug.

However, at the highest concentration assayed, the free drug (**** *p* < 0.0001) and NSs–MPH (* *p* < 0.05) presented a significant difference compared to the medium control. Despite this, it is important to note that there was a significant difference in the mitochondrial activity observed between free MPH and NSs–MPH at 1 mM (*** *p* < 0.001), with the being former 25-fold lower than the latter (2.4% vs. 59.1%).

This is relevant because most alkylating agents, such as MPH, have shown to produce adverse side effects when they are used as free drug, despite their uses as anti-tumoral drugs [20,27,76].

On the other hand, free CUR depicted low cytotoxic effects, being only significantly different from the medium at 1 mM. Moreover, the effects of CUR on mitochondrial activity were reduced when the drug was encapsulated (NSs–CUR), showing no significant differences at any assayed concentration.

**Figure A2:**
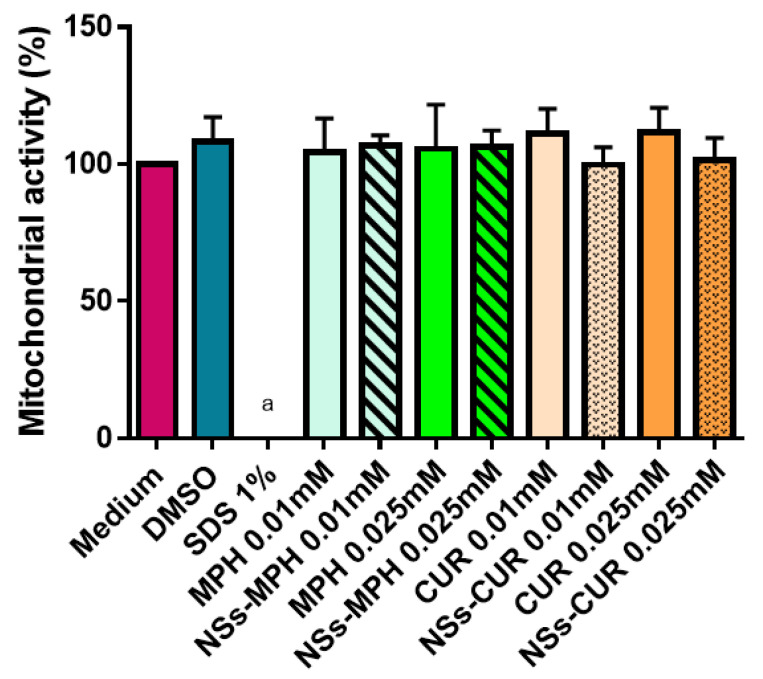
Effects of MPH, CUR, NSs–MPH and NSs–CUR on mitochondrial activity, as determined with the MTS assay. The results are expressed as percentages compared with untreated cells (medium) and represent the mean ± SD of *n* = 3 (a = **** *p* < 0.0001).

Furthermore, we observed that there were not significant effects on HeLa cells when lower concentrations were assayed both for free drugs and their respective complexes forms (Figure A2). These results are very promising because they indicated that the incorporation of MPH or CUR into the NSs significantly reduced their toxic effects on HeLa cells.

Finally, considering the possibility of controlling drug release from the NSs through irradiation (Figure 16), and taking into consideration that the developed nano-formulations proved to be safer in comparison to the free guests (Figure 17), the potential applications of these drug delivery systems are auspicious.

## 4. Conclusions

We successfully included both MPH and CUR inside the cavities of NSs, as proven by ^1^H-NMR, FE-SEM, TEM, DSC, FT-IR, and Raman characterization. The encapsulation percentages were 89% for MPH and 63% for CUR, confirming that NSs can efficiently form an inclusion complex with the drugs. The synthesized AuNRs showed an aspect ratio (length/width) of 6.4, which is consistent with the UV-Vis absorption band in the NIR-II optical window. FE-SEM, EDS, UV-Vis, TEM, DLS and the ζ-potential provided evidence that the NSs–MPH and NSs–CUR systems are appropriate substrates to stabilize AuNRs nanoparticles, as the latter retained their characteristic absorption band in the second biological window (1000–1300 nm), making them suitable for biological and photothermal drug release studies. Cellular studies performed through MTS assays were used to evaluate the inherent cytotoxicity of the drugs before and after encapsulation inside the supramolecular sites of NSs. The mitochondrial activity assays confirmed that the NSs–MPH and NSs–CUR complexes are safer formulations than the free drugs, which is promising in terms of potential biological applications in drug delivery. Finally, via plasmonic heating of the AuNRs associated with the ICs, the ternary systems easily outperformed all the control systems regarding the controlled release of the guests. Drug release percentages were drastically reduced in the control systems in comparison to the ICs–AuNRs after NIR-II irradiation. NSs conjugated to anisotropic gold nanoparticles have been considered in future perspectives, as they are safe, efficient, and non-toxic materials.

## Figures and Tables

**Table 1:** Proton assignments and chemical shifts for NSs, MPH and NSs–MPH.

System	H1	H2	H3	H4	H5	H6	OH2	OH3	OH6
NSs	4.828	3.300	3.628	3.361	3.579	3.655	5.705	5.673	4.440
NSs–MPH	4.825	3.297	3.611	3.359	3.568	3.645	5.720	5.683	4.443
Δδ	0.003	0.003	0.017	0.002	0.011	0.010	−0.015	−0.010	−0.003
**System**	**H′1**	**H′2**	**H′3**	**H′4**	**H′5**	**H′6**			
MPH	3.447	3.738	7.135	6.798	2.835	3.117			
NSs–MPH	3.444	3.733	7.127	6.789	2.828	3.111			
Δδ	0.003	0.005	0.008	0.009	0.007	0.006			

**Table 2:** Proton assignments and chemical shifts for NSs, CUR and NSs–CUR.

System	H1	H2	H3	H4	H5	H6	OH2	OH3	OH6
NSs	4.828	3.300	3.628	3.361	3.579	3.655	5.705	5.673	4.440
NSs–CUR	4.823	3.293	3.609	3.358	3.566	3.644	5.722	5.680	4.445
Δδ	0.005	0.007	0.019	0.003	0.013	0.011	−0.017	−0.013	−0.005
**System**	**H′1**	**H′2**	**H′3**	**H′4**	**H′5**	**H′6**	**H′7**		
CUR	3.835	6.071	6.752	6.798	7.153	7.317	7.544		
NSs–CUR	3.828	6.063	6.743	6.789	7.140	7.301	7.533		
Δδ	0.007	0.008	0.009	0.009	0.013	0.016	0.011		

**Table 3:** DLS, ζ-potential and PDI of AuNRs before and after stabilization with PEG.

System	DLS (Transversal nm)	DLS (Longitudinal nm)	ζ-Potential (mV)	PDI
AuNRs-CTAB	2.5 ± 0.9	46.5 ± 32.9	+33 ± 6.5	0.41
AuNRs-PEG	9.0 ± 4.1	84.7 ± 46.7	−30 ± 3.9	0.47

**Table 4:** DLS, ζ-potential and PDI of NSs–drugs complexes and ICs-AuNRs.

System	DLS (nm)	ζ-Potential (mV)	PDI
NSs	177 ± 15	−37 ± 1.8	0.28
NSs–MPH	243 ± 19	−31 ± 1.3	0.31
NSs–CUR	261 ± 21	−35 ± 1.5	0.38
AuNRs–NSs–MPH	273 ± 27	−22 ± 0.3	0.43
AuNRs–NSs–CUR	288 ± 33	−21 ± 0.7	0.47

**Table 5:** EE% and LC% of the NSs–MPH and NSs–CUR systems.

System	Encapsulation Efficiency (%)	Loading Capacity (%)
NSs–MPH	89.5 ± 0.33	70.1 ± 0.22
NSs–CUR	63.7 ± 0.28	57.7 ± 0.15

**Figure A3:**
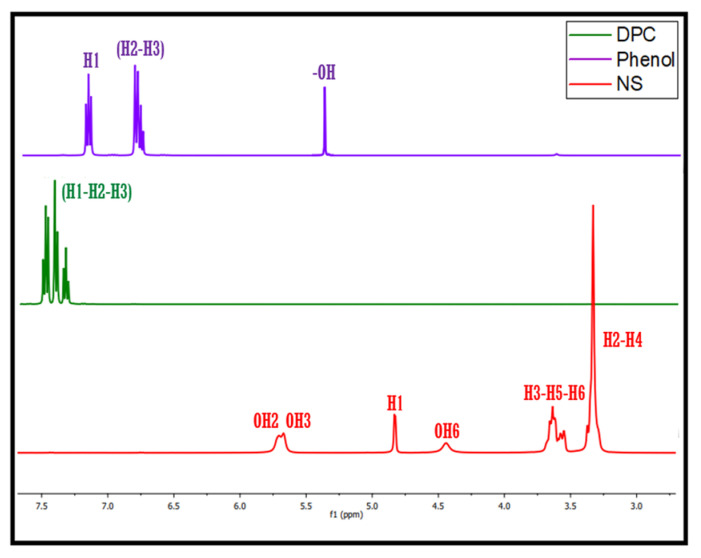
^1^H-NMR (400 MHz, DMSO-d_6_) of NSs, DPC, and phenol.

**Figure A4:**
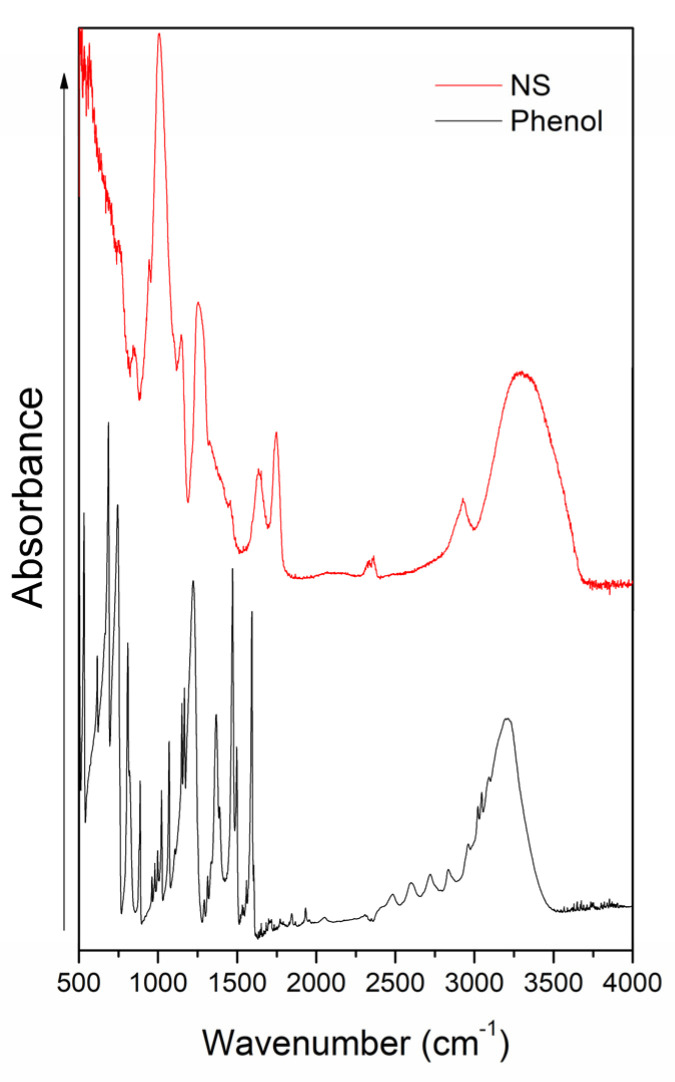
FT-IR spectra of NSs and phenol.

**Figure A5:**
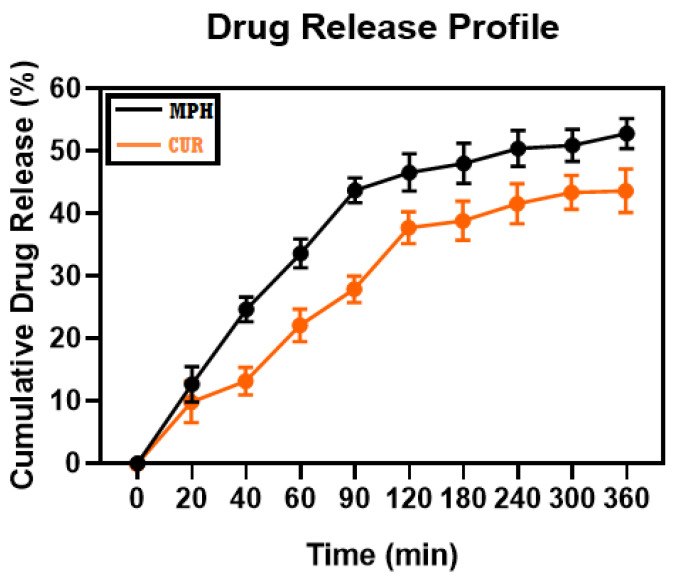
Release profiles of MPH and CUR from the cavities of NSs.

**Figure A6:**
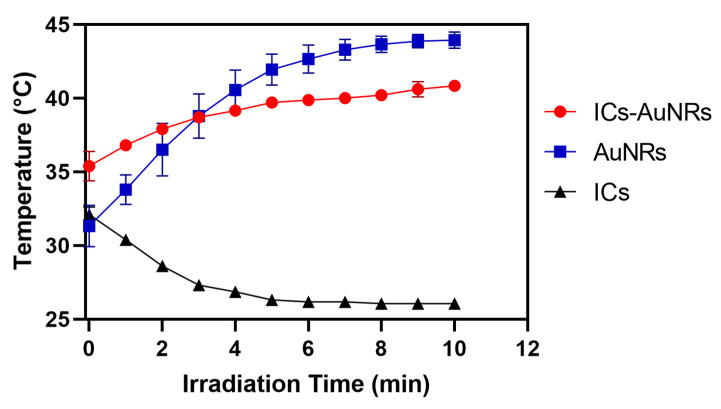
Increase in temperature after irradiating the AuNRs and the ICs–AuNRs systems. The ICs without AuNRs were irradiated as blank.

## Data Availability

Not applicable.

## Appendix A

### Stability of the Ternary Systems after 2 Months of Storage

Colloidal stability of NSs–MPH, NSs–CUR, and the AuNRs–ICs was evaluated after 2 months of storage at 4 °C by means of UV-Vis, DLS, ζ-potential, and PDI. The characteristic plasmon bands, the hydrodynamic diameters and surface charges of the supramolecular systems did not show any significant changes, demonstrating that the formulations maintained their nanometric size, negative ζ-potential and PDI > 0.7 following storage at 4 °C for 2 months, as depicted in Figure A1 and Table A1.

**Table A1:** DLS, ζ-potentials, and PDI values of NSs–drugs complexes and AuNRs–ICs.

System	DLS (nm)	ζ-Potential (mV)	PDI
NSs–MPH	256 ± 21	−30 ± 2.1	0.39
NSs–CUR	277 ± 22	−33 ± 2.3	0.41
AuNRs–NSs–MPH	285 ± 31	−25 ± 1.1	0.52
AuNRs-NSs–CUR	297 ± 37	−27 ± 1.5	0.53

## Appendix C

### Appendix C.1. ^1^H-NMR of NSs, DPC, and Phenol

The removal of by-product phenol and the cross-linker diphenyl carbonate was analyzed using ^1^H-NMR. Figure A3 shows the ^1^H-NMR spectra of NSs, DPC, and phenol. None of the characteristic peaks in DPC (H1, 7.40 ppm; H2, 7.29 ppm; H3, 7.25 ppm) nor phenol (−OH, 5.35 ppm; H1, 7.20 ppm; H2, 6.93 ppm; H3, 6.83 ppm) were observed in the ^1^H-NMR spectra of NSs, thus confirming the elimination of by-products and residual cross-linker. Table A2 shows the proton assignments and the respective chemical shifts for DPC and phenol.

**Table A2:** Proton assignments and chemical shifts for DPC and phenol.

System	H1	H2	H3	−OH
DPC	7.401	7.298	7.253	-
Phenol	7.203	6.933	6.837	5.351

### Appendix C.2. FT-IR of NSs and Phenol

The removal of the phenol was also analyzed using FT-IR. Figure A4 shows the FT-IR spectra of NSs and phenol. The NSs bands were observed at 3388 cm^−1^ (−OH, stretching), 2929 cm^−1^ (C-H stretching), 1030, and 1079 cm^−1^ (C-O stretching), 1368, 1235, and 1155 cm^−1^ (−OH bending), and 1760 cm^−1^ (C=O stretching). The fact that the most characteristic bands ascribed to phenol, namely 3230 cm^−1^ (−OH stretching), 1410, and 1320 cm^−1^ (C-O stretching), were not detected in the NSs spectra suggests that the by-product was removed successfully.

## Appendix D

### Appendix D.1. Swelling Degree of NSs

The swelling studies of NSs were carried out using previously reported methods [77,78,79]. In brief, 100 mg of NSs were immersed in 10 mL of deionized water. Afterward, an aliquot of Na_2_CO_3_ (10% *w*/*w*) was added. After 48 h, a layer of water-bound NSs was obtained by centrifugation. The weight of the hydrated NSs was measured, and the swelling degree was estimated using the following equation:

(A1) $$\mathrm{Sw}\;\left(\%\right)=\frac{\mathrm{NSs}\;\left(h\right)-\mathrm{NSs}\;\left(d\right)\;}{\;\mathrm{NSs}\;\left(h\right)}\times 100\%$$

where NSs (d) is the initial weight of the dry polymer (mg), and NSs (h) is the weight of the swollen sample (mg) at a determined time. Following 48 h, the swelling degree obtained for the NSs synthesized with a (1:4) β-CD: DPC molar ratio was (58.7 ± 6.3%), which is in agreement with previous studies [80].

### Appendix D.2. Solubility Tests of NSs

Solubility tests were conducted to determine whether NSs are soluble or insoluble in organic and inorganic solvents. The results are summarized in Table A3. Increasing aliquots of solvent were added to a fixed amount of NSs (10 mg) at room temperature.

**Table A3:** Solubility of NSs in inorganic and organic solvents.

Solvent	Solubility (25 °C)
Milli-Q water (18 MΩ cm^−1^)	<1 (mg/mL)
Ethanol (≥99.8%)	<5 (mg/mL)
DMSO-d_6_ (99.8%)	10 (mg/mL)
Chloroform-D (99.8%)	<5 (mg/mL)
D_2_O (99.8%)	<1 (mg/mL)
CD_2_Cl_2_ (99.8%)	<1 (mg/mL)

## Appendix E

### Release Profiles of MPH and CUR from the Cavities of NSs

Release profiles of the drugs were analyzed following previously described methods [53,65,78]. Drug formulations containing 1 mg of MPH or CUR were suspended in 3 mL of release medium and sonicated. Then, the NSs–MPH and NSs–CUR complexes were each placed in a dialysis membrane tube, inserted in 100 mL of ethanol: phosphate buffer of pH 7.4 (1:1), and placed in a water bath shaker fixed at 37 °C and 100 rpm. At determined intervals, 1 mL aliquots were changed with an equivalent amount of release medium, replacing hist with fresh medium at a predetermined time. Spectrophotometric analyses were carried out at different times for MPH or CUR content. The release profile studies were performed in triplicate, plotting the mean values as a cumulative percent of released drug versus time. Release profiles of both MPH and CUR are illustrated in Figure A5.

## Appendix F

### Photothermal Efficiency of AuNRs

The photothermal efficiency of AuNRs and the ICs–AuNRs systems are shown in this section, as reported by previous studies involving AuNRs (for further details on the methodology, see references [42,67,81]). Photothermal efficiency indicates the system’s ability to convert incident light into thermal energy. The increase in temperature and the calculated photothermal efficiencies for AuNRs and the ICs–AuNRs are summarized in Figure A6 and Table A4, respectively. The decrease in the photothermal efficiencies in the ICs-AuNRs systems can be ascribed to the effect of the organic matrix in heat dissipation.

**Table A4:** Photothermal efficiencies of AuNRs, ICs–AuNRs, and ICs without AuNRs.

System	T Max (K)	Initial T (K)	ΔT (K)	τs (seg)	Photothermal Efficiency (%)
AuNRs	317.1	304.6	12.5	204.7	69.1
ICs–	314.1	304.3	9.8	227.4	25.3
